# Stratifying the initial prostate cancer suspicion to avoid magnetic resonance exams by sequencing men according to serum prostate‐specific antigen, digital rectal examination and the prostate‐specific antigen density based on digital rectal prostate volume category

**DOI:** 10.1002/bco2.211

**Published:** 2022-12-28

**Authors:** Juan Morote, Marina Triquell, Miriam Campistol, José M. Abascal, Pol Servian, Enrique Trilla

**Affiliations:** ^1^ Department of Urology Vall d'Hebron Hospital Barcelona Spain; ^2^ Department of Surgery Universitat Autòmoma de Barcelona Barcelona Spain; ^3^ Department of Urology Parc de Salut Mar Barcelona Spain; ^4^ Department of Surgery Universitat Pompeu Fabra Barcelona Spain; ^5^ Department of Urology Germans Trias i Pujol Hospital Badalona Spain

**Keywords:** clinically significant, early detection, magnetic resonance imaging, prostate cancer, suspicion

After the U.S. Preventive Task Force recommended against screening for prostate cancer (PCa), the early detection of PCa has moved towards clinically significant PCa (csPCa), reducing the number of unnecessary prostate biopsies and the overdetection of insignificant PCa. This paradigm change is a result of the spread of multiparametric magnetic resonance imaging (mpMRI), which has a high negative predictive value for csPCa and allows guided biopsies to be performed on detected lesions with a prostate imaging‐report and data system (PI‐RADS) score ≥3.^1^ However, suspicion of PCa is still based on serum prostate‐specific antigen (PSA) elevation and/or abnormal digital rectal examination (DRE), and the demand for mpMRI exams has increased to the extent that it is no longer affordable in many centres.^2^ The European Association of Urology (EAU) currently recommends the use of risk‐organised models for the early detection of csPCa, stratifying the initial PCa suspicion to avoid unnecessary mpMRI exams and subsequent biopsies.^2^ At experienced centres, mpMRI will be substituted with biparametric MRI, reducing the scan time fourfold while maintaining the quality and reproducibility of PI‐RADS.^3^ PSA density (PSAD) has been proposed to avoid unnecessary biopsies after mpMRI and also to avoid unnecessary mpMRIs.^4^ It has been proposed that PSA density (PSAD) should be used to avoid unnecessary mpMRIs and subsequent biopsies.^4^ Prostate volume is usually unknown at the time of PCa suspicion; it should therefore be assessed using the DRE category because transrectal ultrasound (TRUS) is not usually performed to assess prostate volume outside of the prostate biopsy procedure.^5^ Roobol et al.^6^ proposed that, where TRUS measurement is not available, DRE should be used to estimate prostate volume for the Rotterdam risk calculator. Additionally, there is evidence that men with PSA > 10 ng/ml and abnormal DRE do not benefit from mpMRI and guided biopsies; these men should therefore be directly scheduled for TRUS systematic prostate biopsies.^7^ We intend to analyse the effectiveness of sequencing the stratification of men with suspected PCa according to their serum PSA levels and DRE, and thereafter according to the PSAD based on their DRE prostate volume category (PSAD‐DRE), to avoid mpMRI exams.

We retrospectively analysed a multicentre dataset of 2881 correlative men with suspected PCa, based on PSA > 3.0 ng/ml and/or abnormal DRE. After pre‐biopsy 3‐Tesla mpMRI, those with lesions with a PI‐RADS v.2 score ≥ 3 were scheduled for 2‐ to 4‐core transrectal ultrasound (TRUS) cognitively guided biopsies and 12‐core TRUS systematic biopsies. Those with a PI‐RADS v.2 score <3 were scheduled for only 12‐core TRUS systematic biopsies. The data were collected between 2016 and 2021 at three academic centres in the metropolitan area of Barcelona. The project was approved by the Vall d'Hebron ethics committee (PRAG‐02/2021). CsPCa, defined as an International Society of Uropathology grade group of 2 or higher, was detected in 1119 men (38.8%). Within a subset of 302 men (10.5%) with serum PSA > 10 ng/ml and abnormal DRE on initial assessment, 264 cases of csPCa (87.4% of the subset) were detected using the systematic biopsies described above.^7^ In the remaining 2579 men, PSAD‐DRE was calculated from the median MRI prostate volume of the corresponding range of DRE prostate volume categories. Category I corresponded to small prostates with less than 30 ml, which were detected in 321 men (12.4%) been 25 ml the median MRI prostate volume. Category I corresponded to small prostates (i.e., <30 ml; median MRI prostate volume 25 ml), which were observed in 321 men (12.4%); category II corresponded to intermediate‐sized prostates (i.e., 30–59 ml; median MRI prostate volume 46 ml), which were observed in 1160 men (45.0%); and category III corresponded to large prostates (i.e. ≥ 60 ml; median MRI prostate volume 82 ml), which were observed in 1098 men (42.6%). The area under the curve analysing the csPCa discrimination of PSAD‐DRE was 0.746 (95% CI: 0.727–0.711), whereas that of PSAD‐MRI was 0.770 (95% CI: 0.753–0.788), *p* = 0.037, Figure 1A. The area under the curve analysing the csPCa discrimination of PSAD‐DRE was 0.746 (95% CI: 0.727–0.711), while that of PSAD‐MRI was 0.770 (95% CI: 0.753–0.788) (*p* = 0.037; Figure 1A). The specificity of PSAD‐DRE, at 95% sensitivity (cut‐off: 0.0577), was 13.9%; for PSAD‐MRI it was 17.6% (cut‐off: 0.0586). The positive and negative predictive values were 36.3% and 87.3%, respectively, for PSAD‐MRI, and 35.4% and 84.8% for PSAD‐DRE. We note that 13.5% and 10.9% of MRI exams and of subsequent prostate biopsies would be avoided using PSAD‐MRI and PSAD‐DRE respectively (*p* < 0.001; Figure 1B). Although, PSAD‐MRI seems better than PSAD‐DRE to predict csPCa, an initial stratification of PCa suspicion by sequencing men from the serum PSA level and DRE and thereafter from the PSAD‐DRE would avoid 584 mpMRI exams (20.3%) compared with 649 (22.6%) when the PSAD‐MRI would be hypothetically used. Importantly, the rate of missed csPCa (3.8%) was the same with both types of PSAD (*p* = 0.028).

**FIGURE 1:**
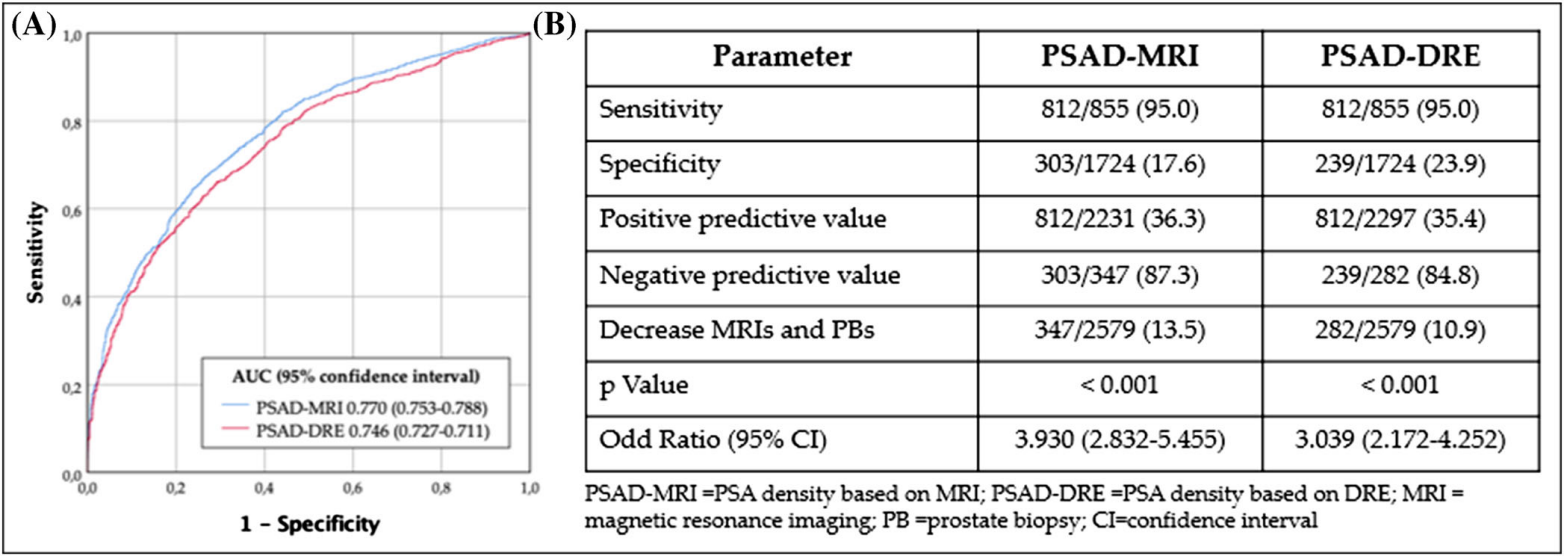
Discrimination analysis of csPCa trough receiver operating characteristic curves of PSAD‐MRI and PSAD‐DRE (A). Performance of PSAD‐MRI and PSAD‐DRE to detect 95% of csPCa, after excluding men with suspected PCa having a serum PSA > 10 ng/ml and abnormal DRE (B)

This study shows how calculations of PSAD made using DRE‐based prostate volume category estimates are slightly less accurate than those made using PSAD‐MRI. However, categorisation using PSA levels and DRE followed by the use of PSAD‐DRE is effective in reducing the demand for mpMRI exams. The proposed approach is in line with the EAU recommendation to promote risk‐based organised models to improve the early detection of csPCa, and 20% of mpMRI exams could be avoided while missing less than 4% of csPCa cases. A recent study found a good correlation between DRE prostate volume assessments and MRI assessments.^8^ The limitations of our study are the lack of studies correlating DRE prostate volume categories with MRI prostate volume ranges for PSAD‐DRE estimation, and the lack of a head‐to‐head comparison between PSAD‐DRE and risk calculators that also use DRE‐based prostate volume categories. To truly ensure the accuracy of any test, a gold‐standard histopathological test such as template saturation biopsy or radical prostatectomy should be used, rather than the currently recommended biopsy approach. Finally, we wish to note that our findings vindicate the use of DRE: When applied in a systematic manner, as reported here, this somewhat forgotten technique is capable of adequately establishing PCa suspicion. DRE provides a variety of valuable information: abnormalities on the posterior surface of the prostate gland, which are suggestive of PCa, can be detected, prostate volume can be categorised, and the presence of inflammation can be inferred from patient‐reported pain upon digital pressure of the prostate surface. New studies should validate our results to be implemented as risk‐organised models of csPCa.

## CONFLICT OF INTEREST

The authors have no conflict of interest to declare.

## AUTHOR CONTRIBUTIONS

J. M. conceptualised the idea. M. T., M. C., J. M. A., P. S. and E. T. developed the concept. J. M. wrote the first draft of the manuscript. All authors were involved in editing, critical review and final approval of the manuscript.
